# Violent video games exposure and aggression: The role of moral disengagement, anger, hostility, and disinhibition

**DOI:** 10.1002/ab.21860

**Published:** 2019-08-22

**Authors:** Mengyun Yao, Yuhong Zhou, Jiayu Li, Xuemei Gao

**Affiliations:** ^1^ Faculty of Psychology Southwest University Chongqing China; ^2^ Key Laboratory of Cognition and Personality, Ministry of Education Southwest University Chongqing China

**Keywords:** aggressive behavior, anger, disinhibition, hostility, moral disengagement, violent video games exposure

## Abstract

Based on the General Aggression Model (GAM), the current study investigated the interactive effect of personal factors (e.g., sensation‐seeking) and situational factors (e.g., violent video games exposure [VVGE]) on the trait aggressive behavior, and the mediating role of individual difference trait (e.g., moral disengagement, anger, and hostility). We recruited 547 undergraduates (48.45% male) from five Chinese universities. The results showed that VVGE was positively associated with moral disengagement, disinhibition, and the four aggressive traits (physical aggression, verbal aggression, anger, and hostility), which were positively associated with each other. Moral disengagement was positively associated with both the disinhibition and the four aggressive traits. Disinhibition was positively associated with the four aggressive traits as well. When controlled for gender, moral disengagement, anger, and hostility wholly mediated the relationship between VVGE and aggression, but the moderation effect of disinhibition was not significant. These findings support the framework of GAM and indicate that moral disengagement, anger, and hostility may be the factors that increase the risk of a higher level of aggression following repeated exposure to violent video games.

## 1 INTRODUCTION

Player Unknown's Battlegrounds (PUBG), a shooting game that Chinese players call “chicken dinner”, has recently become popular among young people, quickly overtaking Honor of Kings in terms of popularity. According to the China gaming industry report from January to June 2018, the top two games for sales in the mobile video games market were Action Role Playing Game (29.9%) and Multiplayer Online Battle Arena (MOBA; 17.4%), which accounted for nearly 50% of sales, and the proportion of Shooting Games has also increased significantly. Furthermore, the report showed that 35.9% of the game types were Shooting Games and 17.9% were MOBA in the Chinese client e‐sports game market (China Audio‐video & Digital Publishing Association Game Publishing Committee, 2018). Many games of such genres (e.g., PUBG) contain violent content (Teng, Li, & Liu, 2014), which explains to a certain extent the universality of violent video games.

Violent video games are those that depict intentional attempts by individuals (nonhuman cartoon characters, real persons, or anything in between) to inflict harm on others (Anderson & Bushman, 2001). The effects of violent video games have been a societal concern since the birth of the industry and have attracted much attention from researchers. A large body of research has found that violent video game exposure (VVGE) is associated with increased aggression among individuals at various ages (e.g., Gentile, Bender, & Anderson, 2017; Greitemeyer, 2018; Krahé, 2014; Teng et al., 2019; Velez, Greitemeyer, Whitaker, Ewoldsen, & Bushman, 2016). Also, some research has examined the pathways in the associations between VVGE and aggression; for instance, mediators such as hostile attribution bias, aggressive norms, and dehumanization (e.g., Anderson, Gentile, & Buckley, 2007; Gentile, Li, Khoo, Prot, & Anderson, 2014; Greitemeyer & McLatchie, 2011; Möller & Krahé, 2009), and moderators such as psychoticism, aggressive traits, neuroticism, and conscientiousness (e.g., Markey & Markey, 2010; Markey & Scherer, 2009). To the best of our knowledge, however, there have been few studies that have examined simultaneously the underlying mechanisms of the link between VVGE and aggression from the perspectives of social cognition (i.e., moral disengagement) and personality trait (i.e., sensation seeking, anger, hostility). Such a comprehensive study could help to develop interventions to reduce the relation between VVGE and aggressive behaviors from a theoretical perspective.

### 1.1 Violent video games exposure and aggression

Although some recent studies have not found a significant relationship between VVGE and aggression (Ferguson & Kilburn, 2010; McCarthy, Coley, Wagner, Zengel, & Basham, 2016; Pan, Gao, Shi, Liu, & Li, 2018), a relatively solid association has been established in experimental, cross‐sectional, and longitudinal studies in general. For example, most research in this area has found that violent video games increase aggressive thoughts, angry feelings, physiological arousal, and aggressive behaviors, and decrease empathic feelings and helping behaviors (e.g., Anderson et al., 2010; Gentile et al., 2017; Hasan, Bègue, & Bushman, 2012; Verheijen, Burk, Stoltz, Van, & Cillessen, 2018). In addition, some research in cognitive neuroscience has provided neuroimaging support for these effects (e.g., Gentile, Swing, Anderson, Rinker, & Thomas, 2016; Montag et al., 2012), and there are also meta‐analyses that have concluded that violent video games increase aggression (e.g., Bushman, 2016; Greitemeyer & Mügge, 2014).

How does VVGE affect individual aggression? The General Aggression Model (GAM), a general model to account for aggressive behavior, could answer this question. GAM consists of two major systems: personality development (distal processes) and social encounters (proximate processes). The proximate processes explain individual episodes of aggression using three stages, that is, personal and situational inputs influence internal states (cognition, affect, and arousal), which in turn affect appraisal and decision processes, which in turn influence aggressive and nonaggressive behavioral outcomes. Each cycle of the proximate processes serves as a learning trail that creates aggressive knowledge structures after many repetitions. Distal processes detail how biological and persistent environmental factors influence personality through changes in knowledge structures (aggressive beliefs and attitudes, aggressive perceptual schemata, aggressive expectation schemata, aggressive behavioral scripts, and aggression desensitization) and brain structure and function. The personality, in turn, influences personal and situational factors in a cyclical fashion (Allen, Anderson, & Bushman, 2018; Anderson & Bushman, 2002; Anderson & Bushman, 2018). VVGE has been assumed to be a situational input variable of proximal causal factors and an environmental factor of distal causal factors (Anderson & Bushman, 2018), that is, VVGE influences aggression through the two main systems of GAM.

Most violent video games primarily involve physical violence, and many of the multiplayer games also involve verbal violence (Adachi & Willoughby, 2016; Lemmens, Valkenburg, & Peter, 2011), therefore, we focused on self‐reported forms of physical aggression and verbal aggression in the current study.

### 1.2 Moral disengagement as a potential mediator

Moral disengagement is a cognitive predisposition that individuals reinterpret their immoral behaviors (Bandura, Barbaranelli, Caprara, & Pastorelli, 1996). In general, individuals have their own moral standards that inhibit them from engaging in immoral conduct (Bandura, 1990), but these standards can be deactivated selectively through eight moral disengagement mechanisms (Bandura, 1999). Thus, an individual's moral disengagement mechanisms may be exerted when they commit aggressive acts.

Previous research has supported the moral disengagement theory that moral disengagement mechanisms can make individuals reconstruct aggression cognitively; thus aggression is more likely to occur (Bandura et al., 1996). For instance, numerous cross‐sectional studies have found that moral disengagement is positively associated with various forms of aggressive behavior such as physical aggression, verbal aggression, and bullying (e.g., Bussey, Quinn, & Dobson, 2015; Gao, Weng, Zhou, & Yu, 2017; Obermann, 2011; Rubio‐Garay, Carrasco, & Amor, 2016). Also, this correlation was found to be significant in juvenile delinquent samples (Wang, Lei, Yang, Gao, & Zhao, 2016; Zapolski, Banks, Lau, & Aalsma, 2018). Moreover, longitudinal studies have found that initial moral disengagement can predict later aggression among adolescents (e.g., Barchia & Bussey, 2011; Hyde, Shaw, & Moilanen, 2010; Paciello, Fida, Tramontano, Lupinetti, & Caprara, 2008; Sticca & Perren, 2015). In addition, a recent meta‐analysis has reinforced this link (Gini, Pozzoli, & Hymel, 2014; Killer, Bussey, Hawes, & Hunt, 2019).

Moral disengagement is not only a powerful predictor of aggression but also a product of VVGE. Some longitudinal research has established a stable link between the two, indicating that frequent exposure to violent video games in early sessions can predict higher levels of moral disengagement in later sessions; however, this effect was not found to be significant when the position of these two variables was reversed (Teng, Nie, Pan, Liu, & Guo, 2017; Wang, Ryoo, Swearer, Turner, & Goldberg, 2017). In addition, some cross‐sectional studies have also found an association between VVGE and higher levels of moral disengagement (Gabbiadini, Andrighetto, & Volpato, 2012; Teng, Nie, Guo, & Liu, 2017).

As mentioned above, moral disengagement may be a potential mediator in the relationship between VVGE and aggression. Richmond and Wilson (2008) found that the relationship between violent media exposure frequency and aggression was mediated wholly by moral disengagement. As for violent video games in particular, research has found that dehumanization, one of the moral disengagement mechanisms, mediates the effect of VVGE on aggressive behavior (Greitemeyer & McLatchie, 2011). Teng et al. (2019) further demonstrated through a longitudinal study that moral disengagement mediates the link between VVGE and aggression, especially for early adolescents. However, as the research‐tested adolescents from the ages of 12–19 years, it is unclear whether the results can be generalized to adults.

Our research aimed to further test the role of moral disengagement in the relationship between VVGE and aggression among college students. Based on the literature reviewed above, it is reasonable to expect that moral disengagement would play a mediating role in the relationship. Thus we propose the following hypothesis:

**H1**: *Moral disengagement will play a mediating role in the relationship between VVGE and aggression.*

### 1.3 Anger and hostility as potential mediators

Anger involves physiological arousal and preparation for aggression, representing the emotional or affective component of behavior, and hostility consists of feelings of ill will and injustice, representing the cognitive component of behavior (Buss & Perry, 1992). Research has explored the relationship between VVGE, anger, hostility, aggression, as follows. Anger moderated the relationship between VVGE and aggression (Engelhardt, Bartholow, & Saults, 2011; Giumetti & Markey, 2007), hostility mediated the relationship between VVGE and aggression (Adachi & Willoughby, 2016; Bartholow, Sestir, & Davis, 2005; Gentile, Lynch, Linder, & Walsh, 2004). But according to GAM, anger, and hostility may also be potential mediators.

According to the short‐term effects (proximal processes) of GAM, violent video gameplay, when combined with a provocation, may increase anger and hostility, thereby increasing the likelihood of subsequent aggressive behavior. The long‐term effects of GAM (distal processes) suggest that repeated exposure to violent video games changes aggressive knowledge structures, and finally contributing to enhanced aggressive personality (Anderson & Bushman, 2002; Anderson & Bushman, 2018). Rather trait anger and trait hostility are cognition correlated knowledge structures (Anderson & Bushman, 2001; Anderson et al., 2010). Therefore, according to GAM, anger, and hostility may be potential mediators. Thus, we propose the following hypothesis:

**H2**: *Anger and Hostility will play a mediating role in the relationship between VVGE and aggression.*

### 1.4 Disinhibition as a potential moderator

Although VVGE has a significant effect on aggression, not all individuals are affected by VVGE in equal measure. Research has found that users with particular characteristics are more susceptible to VVGE effects than others (Exelmans, Custers, & Van den Bulck (2015); Markey & Markey, 2010; Markey & Scherer, 2009). According to the GAM, the interactive dynamics of personal and situational (i.e. VVGE) factors, of biological and environmental (i.e. VVGE) factors will influence an individual's aggressive behaviors. Based on this theory, users’ characteristics such as personality traits could moderate the association between VVGE and aggression.

Previous research has found that callous‐unemotional traits, psychoticism, aggressive traits, and empathy could moderate the relationship between VVGE and aggression (Gao et al., 2017; Krahé & Möller, 2010; Markey & Scherer, 2009; Rydell, 2016). As another form of personality trait, sensation‐seeking may also serve as a moderator between VVGE and aggression. Sensation seeking is defined by the seeking of varied, novel, complex and intense sensations and experiences, and the willingness to take physical, social, legal and financial risks for the sake of such experiences (Zuckerman, 1994). Sensation seeking has been identified as a moderator of the relationship between violent media content and aggression (Slater, Henry, Swaim, & Cardador, 2004). However, Bisch and Lee (2009) found that the interaction effect between violent video games and sensation seeking was not significant. Sensation seeking contains four subscales: thrills and adventure‐seeking; experience seeking; disinhibition; and boredom susceptibility. It may be that particular dimensions are the main factors in the effect of sensation seeking as a moderator.

The disinhibition dimension may be qualitatively different from the other three dimensions (Krcmar & Greene, 1999). Disinhibition represents the desire for social and sexual disinhibition as expressed in social drinking, partying, and variety in sexual partners (Zuckerman, 1994). It is the reverse of inhibition and describes how people reduce their public self‐awareness, have less concern about the judgment of others, and thus ignore conventional constraints (Lin & Tsai, 2002). Research has found that the disinhibition dimension and the experience‐seeking dimension are related to adolescents’ exposure to violent television positively and negatively, respectively (Krcmar & Greene, 1999). Additionally, Aluja‐Fabregat (2000) found a positive relation between disinhibition and exposure to violent films in 8th‐grade boys and girls. Moreover, a recent study that compared gamers (former and ongoing) with non‐gamers found an association between disinhibition and VVGE (Kimmig, Andringa, & Derntl, 2018). Consequently, it seems that disinhibition is the main factor in the moderation of the relationship between VVGE and aggression via sensation seeking.

However, although research has identified sensation seeking as a moderator in the relationship between violent media use and aggression, some studies have not found this effect with regard to VVGE. Given the findings cited above, it is reasonable to deduce that the disinhibition dimension may play a different role in the relationship between VVGE and aggression. Thus we propose the following hypothesis:

**H3**: *Disinhibition will moderate the relationship between violent video games exposure and aggression.*

### 1.5 The present study

The aims of the present study were twofold: first, we aimed to examine the mediating effect of moral disengagement, anger, and hostility in the relationship between VVGE and aggression among college students. Second, we aimed to examine whether disinhibition dimension of sensation seeking plays a role as a moderator between VVGE and aggression. These two questions can address the mechanisms of both mediation (i.e., how does VVGE increase aggression), and moderation (i.e., when and for whom is the effect most potent) of the relationship between VVGE and aggression.

## 2 METHOD AND MATERIALS

### 2.1 Participants

The present study used convenient cluster sampling technology to recruit 855 college students from five universities in China as participants, based on the accessibility. We recovered 757 surveys, and among them were 547 valid responses (excluding incomplete surveys and false answers). The final sample included 265 males and 282 females. The participants’ ages ranged from 16 to 26 years (*M *=* *19.34; *standard deviation *=* *1.01).

### 2.2 Measures

#### 2.2.1 Video game questionnaire

To measure VVGE, we used the video game questionnaire adapted by Gentile et al. (2004) from Anderson and Dill (2000). Participants were asked to list their three favorite video games, including any games played on computers, video game consoles, hand‐held devices, or in video arcades. They were also asked to record the frequency of their play on a 7‐point scale for each game (1* *=* *“rarely”, 7* *=* *“often”). They then rated the extent of the violence of each game's content and graphics on a 7‐point scale (1* *=* *“little or no violence”, 7* *=* *“extremely violent”). The average rating of the video games was used as the overall index of the VVGE. The index was calculated as: ∑[(the content rating + the graphics rating) × (the weekday frequency × 5 + the weekend frequency × 2)] ÷ the number of games. And participants who never played video games were given a VVGE score of one. The higher the score is, the higher the level of VVGE will be. In the present study, Cronbach's α for the scale is 0.83.

#### 2.2.2 Moral disengagement scale (MDS)

The MDS was used to measure moral disengagement (Bandura et al., 1996). The Chinese version has been demonstrated to be a reliable and valid measurement (Yang & Wang, 2012). The scale includes 32 items divided into eight mechanisms: moral justification, euphemistic language, advantageous comparison, displacement of responsibility, diffusion of responsibility, distorting consequences, attribution of blame, and dehumanization. All items use a 5‐point scale (1* *=* *“strongly disagree”, 5* *=* *“strongly agree”), and higher total scores indicate higher levels of moral disengagement. In the present study, Cronbach's α for the scale is 0.94.

#### 2.2.3 Buss–Perry aggression questionnaire (BPAQ)

The BPAQ consists of 29 items, divided into four dimensions: physical aggression, verbal aggression, anger, and hostility (Buss & Perry, 1992). All items use a 5‐point scale (1 = “strongly disagree”, 5 = “strongly agree”). The Chinese version of BPAQ has high validity and reliability (Wang et al., 2016). In the present study, Cronbach's α for the scale is 0.91.

The present study used the physical aggression and verbal aggression subscales to assess the trait aggressive behavior, and anger and hostility subscales to access the trait anger and trait hostility. Higher scores indicate higher aggression trait, respectively. In the present study, Cronbach's α for the physical aggression subscale is 0.81, verbal aggression subscale is 0.74, anger subscale is 0.83; hostility subscale is 0.80.

#### 2.2.4 Sensation‐seeking scale (SSS‐V)

The SSS‐V (Zuckerman, Eysenck, & Eysenck, 1978) consists of 40 items based on forced choice. Participants choose one statement from two options that best describes them and receive one point for each choice that corresponds to sensation seeking. The Chinese version of the SSS‐V (Wang et al., 2000) shows good validity and reliability and has been widely used. In the present study, Cronbach's α for the sensation‐seeking scale is 0.61. The study used the disinhibition subscale to measure disinhibition; higher disinhibition scores represent higher disinhibition tendencies. Cronbach's α for the disinhibition subscale is 0.52, higher disinhibition scores represent higher disinhibition tendencies.

### 2.3 Procedure and data analysis

The study was approved by the researchers’ University Ethics Committee. Before the investigation, all participants were told that the study was being conducted anonymously and that their information would remain confidential. We then obtained informed consent and participants completed the questionnaires, guided by trained researchers. All the participants were voluntary and they were free to withdraw from the study at any time.

Descriptive statistics, gender differences, correlation analysis, and regression analysis of main variables were conducted using SPSS 22.0. The mediation and moderation analysis was carried out using PROCESS macro (Hayes, 2013). The bootstrapping method (Hayes, 2013; Preacher & Hayes, 2004), which can attain robust standard errors for parameter estimation, was used to test the significance of the mediating effect and moderating effect. We set 5,000 bootstrapping samples and 95% bias‐corrected confidence intervals (CI). Cl containing zero indicated significant effects.

## 3 RESULTS

### 3.1 Preliminary analyses

The study used a self‐report design to collect data, which meant that common method variance may have existed. We used Harman's single‐factor test to test the common method bias. The test showed that there were 36 factors with eigenvalues greater than one, which together explained 65.24% of the total variance, with the largest single factor explaining 14.23% of the variance, which is less than the judgment standards of 40% (Podsakoff, Mackenzie, Lee, & Podsakoff, 2003). Therefore, the common method bias was not problematic in this study.

Table 1 shows the correlations between the main variables with gender dummy coded. VVGE was positively associated with moral disengagement, disinhibition, and the four aggressive traits, which were positively correlated with each other. Moral disengagement was positively associated with both the disinhibition and the four aggressive traits. Disinhibition was positively associated with the four aggressive traits. Gender, as a covariate in subsequent analyses, was positively associated with every variable except trait anger.

**Table 1:** Correlations and means of study variables

	*M*	*Standard deviation*	1	2	3	4	5	6	7	8
1 VVGE	74.95	64.37	1							
2 Physical aggression	18.51	6.10	0.30***	1						
3 Verbal aggression	12.85	3.71	0.22***	0.54***	1					
4 Anger	15.93	5.34	0.19***	0.61***	0.53***	1				
5 Hostility	19.29	5.57	0.16***	0.52***	0.47***	0.63***	1			
6 Moral disengagement	67.41	20.54	0.29***	0.51***	0.36***	0.31***	0.41***	1		
7 Disinhibition	3.53	1.89	0.19***	0.31***	0.10*	0.11*	0.14**	0.31***	1	
8 Gender			0.39***	0.35***	0.16***	0.03	0.10*	0.43***	0.33***	1

Abbreviation: VVGE, violent video games exposure.

* *p *< .05.

** *p *< .01.

*** *p *< .001.

### 3.2 The mediating effect of moral disengagement, anger, and hostility

**Figure 1:**
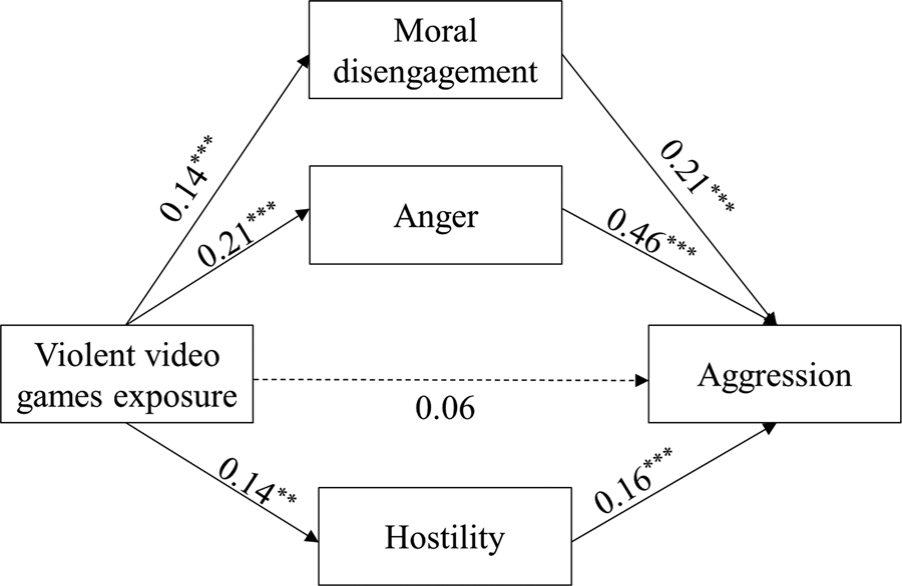
The relationship between VVGE, moral disengagement, anger, hostility, and aggressive behavior. VVGE, violent video games exposure

To test Hypothesis 1 and Hypothesis 2 that moral disengagement, anger, and hostility would mediate the relationship between VVGE and aggression, we conducted the PROCESS macro Model 4 of SPSS (Hayes, 2013) with all data standardized. In the model, VVGE was entered as the predictor, moral disengagement, anger, and hostility as the mediators, aggressive behavior (the composite of physical aggression and verbal aggression) as the outcome variable, and gender was included as a covariate. The mediation effects of moral disengagement (0.03), anger (0.10), and hostility (0.02) were significant (see Table 2, Table 3, and Figure 1). Moral disengagement, anger, and hostility accounted for 14.29, 47.62, and 9.52% of the total effect, respectively. When controlling for moral disengagement, anger, and hostility, the direct effect of VVGE on aggression was not significant (*β *= 0.06; *standard error *= 0.03; 95% CI = [−0.001, 0.12]). Moral disengagement, anger, and hostility wholly mediated the relationship between VVGE and aggression with 71.43% of the total effect.

**Table 2:** Testing the mediation effect of violent video games exposure on aggression (standardized coefficient)

	Predictors	*R* ^*2*^	*F*	*β*	*t*	95% CI
Model 1	VVGE	0.20	67.94***	0.14	3.46***	(0.06, 0.23)
(Moral disengagement)	Gender			0.74	8.90***	(0.58, 0.91)
Model 2	VVGE	0.04	10.58***	0.21	4.55***	(0.12, 0.30)
(Anger)	Gender			−0.10	−1.12	(−0.28, 0.08)
Model 3	VVGE	0.03	7.87***	0.14	3.11**	(0.05, 0.23)
(Hostility)	Gender			0.10	1.05	(−0.08, 0.28)
Model 4	VVGE	0.57	145.30***	0.06	1.95	(−0.001, 0.12)
(Aggressive behavior)	Moral disengagement			0.21	6.24***	(0.15, 0.28)
	Anger			0.46	12.69***	(0.39, 0.54)
	Hostility			0.16	4.36***	(0.09, 0.24)
	Gender			0.32	4.91***	(0.19, 0.45)

Abbreviation: CI, confidence interval; VVGE, violent video games exposure.

** *p *< .01.

*** *p *< .001.

**Table 3:** The direct effect and the mediation effect of moral disengagement, anger, and hostility

	*ab*	*Standard error*	95% CI
Mediation effect 1 (moral disengagement)	0.03	0.01	(0.01, 0.06)
Mediation effect 2 (anger)	0.10	0.03	(0.05, 0.15)
Mediation effect 3 (hostility)	0.02	0.01	(0.01, 0.05)
Total indirect effect	0.15	0.03	(0.08, 0.22)
Direct effect	0.06	0.03	(−0.001, 0.12)

Abbreviation: CI, confidence interval; ab, the mediation effect.

### 3.3 The moderating effect of disinhibition

To test Hypothesis 3 that disinhibition would moderate the relationship between VVGE and aggression, we conducted the PROCESS macro Model 1 of SPSS with disinhibition as a moderator, VVGE as the predictor, aggressive behavior as the outcome variable, gender as a covariate (Hayes, 2013). The results showed that the moderation effect of disinhibition was not significant (*β *= −0.04, *t *= −0.90, 95% CI = [−0.12, 0.04]), see Table 4.

**Table 4:** Testing the moderation effect of violent video games on aggression (standardized coefficient)

Outcome	Predictors	*R* ^*2*^	*F*	*β*	*t*	95% CI
Aggressive behavior	VVGE	0.16	25.41***	0.21	4.75***	(0.12, 0.30)
	Disinhibition			0.16	3.85***	(0.08, 0.24)
	VVGE × disinhibition			−0.04	−0.90	(−0.12, 0.04)
	Gender			0.35	3.94***	(0.18, 0.53)

Abbreviation: CI, confidence interval; VVGE, violent video games exposure.

*** *p *< .001.

## 4 DISCUSSION

Consistent with H1, our study found that moral disengagement played a mediating role in the relationship between VVGE and aggression, suggesting that college students with high levels of VVGE are more likely to use moral disengagement mechanisms, further resulting in enhanced aggressive behavior trait. This finding is consistent with the research of Teng et al. (2019), indicating that the mediation effect of moral disengagement can be generalized to adult college students. The result also adds support for the GAM by the indication that VVGE influences an individual's internal state of cognition—specifically, the cognitive predisposition of moral disengagement (Bandura et al., 1996)—and ultimately an individual's level of aggression (Anderson, & Bushman, 2002; Anderson, & Bushman, 2018).

Each of the separate links in the mediation model is noteworthy. VVGE was positively associated with moral disengagement, the first stage of the mediation process, and this result is consistent with previous research (e.g., Gabbiadini et al., 2012; Greitemeyer & McLatchie, 2011). Teng et al. (2017) explained this result by the use of Bandura's social cognitive theory; that is, VVGE as a contextual variable influences an individual's moral values and cognition, including moral disengagement (Bandura, 2001). Moral disengagement was positively associated with aggressive tendencies, the second stage of the mediation process, and this adds support for previous research (e.g., Paciello et al., 2008; Wang et al., 2016). Bandura's moral disengagement theory proposes that the eight moral disengagement mechanisms can encourage individuals to reconstruct aggression cognitively (e.g., by making the outcome of their behavior appear less harmful; by minimizing their role in the outcome; and by reducing their recognition for the victim), thus aggression is more likely to occur (Bandura et al., 1996). Shu, Gino, and Bazerman (2011) suggest that moral disengagement influences anticipatory guilt reactions, prosocial tendencies, and cognitive and affective reactions; effects that are conducive to immoral or antisocial behavior, such as aggression.

Consistent with H2, our study found that anger and hostility mediated the relationship between VVGE and aggression, suggesting that high level of VVGE is associated with increased anger and hostility in college students, which finally resulted in enhanced aggressive behavior trait. This is in line with the findings from some previous work (Adachi & Willoughby, 2016; Bartholow et al., 2005; Gentile et al., 2004). The result supports the long‐term effects (distal processes) of GAM (Anderson & Bushman, 2002; Anderson, & Bushman, 2018), that repeated VVGE over longer periods of time leads to elevations in more stable aggressive traits (trait anger, trait hostility), and such traits are part of aggression‐related knowledge structures. Finally, the reinforced knowledge structures contribute to the enhancement of aggressive personality, which further influence individuals’ decision together with situational variables.

With regard to H3, our study found that the moderation role of disinhibition, a dimension of sensation seeking, between VVGE and aggression was not significant. Disinhibition represents stimulation seeking through experiences with other individuals, using substances to feel disinhibited, and living a “hedonistic lifestyle” (Wilson & Scarpa, 2014). The characteristics of violent video games provide users with an opportunity for obtaining such experiences above. First, many violent video games are now large online multirole cooperative games, making them a kind of collective activity. Then, violent video games are full of violent and bloody content with immediate reinforcement (Teng et al., 2014) whilst a player can be anonymous; characteristics that make playing such games an unrestricted activity. Players of violent video games can do anything they want and perform acts that they cannot do in real life. And in this process, players are in an excited state with increased physiological arousal (Anderson et al., 2010); that is, through violent video gameplay, players can feel disinhibited and live a hedonistic lifestyle. These considerations help to explain the strong association between violent video games and disinhibition, but our results suggest that disinhibition is not the main factor in sensation seeking to moderate the relationship between VVGE and aggression. It may be due to the low reliability of sensation seeking scales and the disinhibition subscales. Actually, a few college students said they could not make a decision between some forced choices, because they never experienced some activities on the scale. Besides, some activities are forbidden (such as drugs) and some activities are not suitable to be discussed in public (such as sex) in China. So some items may not adapt to Chinese society situation and should be localized first. Or other materials to measure sensation seeking and inhibition should be considered.

The present study expands previous research by generalizing the mediation effect of moral disengagement to adult college students and exploring trait anger and trait hostility as the mediators in the relationship between VVGE and aggression. The results also add support for the social cognitive theory and the GAM to a certain extent. Reducing exposure to violent video games and the probability of moral standards being deactivated (Teng et al., 2019) may be an effective intervention to reduce aggression.

However, the study has several limitations. First, the datasets were collected through cross‐sectional methods, and this limits the inference of causal relationships. Longitudinal research should be conducted in the future. Second, we used self‐report questionnaires to gather the data. Although the common method bias was not problematic, as shown in the preliminary analysis, social desirability bias may exist. Moreover, players with higher levels of moral disengagement or aggression may evaluate the violence level of games lower than their counterparts. Future research could collect data from multiple informants and explore mediation and moderation effects through experimental research. Third, the research methods and sample (using only five universities in southwest China) may have influenced the size of the effects; selecting a more representative sample or improving the research methods may help to increase the size of the effects.

## CONFLICTS OF INTEREST

The authors declare that they have no conflict of interest.

## Supporting information

Supporting information
