# Supplementary material for: Violent video games exposure and aggression: The role of moral disengagement, anger, hostility, and disinhibition
Source: Aggress Behav. 2019 Aug 22;45(6):662–70. doi: 10.1002/ab.21860 (PMC6790562; doi:10.1002/ab.21860)
Supplement: Supplementary file 1 — Supporting information [file AB-45-662-s001.docx]

Supplementary material for review

**1. The analysis of physical aggression**

We conducted the PROCESS macro Model 4 of SPSS (Hayes, 2013) with all data standardized. In the model, VVGE was entered as the predictor, moral disengagement, anger and hostility as the mediators, physical aggression as the outcome variable, and gender was included as a covariate. The mediation effects of moral disengagement (0.03), anger (0.09) and hostility (0.02) were significant (see Table 2, Table 3). Moral disengagement, anger and hostility accounted for 15.79%, 47.37%, and 10.53% of the total effect respectively. When controlling for moral disengagement, anger and hostility, the direct effect of VVGE on aggression was not significant (*β* = 0.05, *SE* = 0.03, 95% *CI* = [-0.02, 0.11]). Moral disengagement, anger and hostility wholly mediated the relationship between VVGE and aggression with 73.68% of the total effect.

**Table 2**. Testing the mediation effect of violent video games exposure on physical aggression (standardized coefficient)

|  | Predictors | *R^2^* | *F* | *β* | *t* | 95% *CI* |
| --- | --- | --- | --- | --- | --- | --- |
| Model 1 | VVGE | 0.20 | 67.94^***^ | 0.14 | 3.46^***^ | (0.06, 0.23) |
| (Moral disengagement) | Gender |  |  | 0.74 | 8.90^***^ | (0.58, 0.91) |
| Model 2 | VVGE | 0.04 | 10.58^***^ | 0.21 | 4.55^***^ | (0.12, 0.30) |
| (Anger) | Gender |  |  | -0.10 | -1.12 | (-0.28, 0.08) |
| Model 3 | VVGE | 0.03 | 7.87^***^ | 0.14 | 3.11^**^ | (0.05, 0.23) |
| (Hostility) | Gender |  |  | 0.10 | 1.05 | (-0.08, 0.28) |
| Model 4 | VVGE | 0.53 | 122.27^***^ | 0.05 | 1.44 | (-0.02, 0.11) |
| (Physical aggression) | Moral disengagement |  |  | 0.22 | 6.01^***^ | (0.15, 0.29) |
|  | Anger |  |  | 0.44 | 11.52^***^ | (0.37, 0.52) |
|  | Hostility |  |  | 0.13 | 3.28^**^ | (0.05, 0.21) |
|  | Gender |  |  | 0.41 | 5.96^***^ | (0.28, 0.55) |

Note: VVGE = Violent video games exposure, ^*^*p*<0.05, ^**^*p* <0.01, ^***^*p* <0.001

**Table 3.** The direct effect and the mediation effect of moral disengagement, anger and hostility

|  | *ab* | *SE* | 95% *CI* |
| --- | --- | --- | --- |
| Mediation effect 1 (Moral disengagement) | 0.03 | 0.01 | (0.01, 0.06) |
| Mediation effect 2 (Anger) | 0.09 | 0.03 | (0.05, 0.14) |
| Mediation effect 3 (Hostility) | 0.02 | 0.01 | (0.01, 0.04) |
| Total indirect effect | 0.14 | 0.03 | (0.08, 0.20) |
| Direct effect | 0.05 | 0.03 | (-0.02, 0.11) |

We conducted the PROCESS macro Model 1 of SPSS with disinhibition as a moderator, VVGE as the predictor, physical aggression as the outcome variable, gender as a covariate (Hayes, 2013). The results showed that the moderation effect of disinhibition was not significant (*β* = -0.03, *t* = -0.81, 95% *CI* = [-0.11, 0.05]), see Table 4.

**Table 4**. Testing the moderation effect of violent video games on aggression

| Outcome | Predictors | *R^2^* | *F* | *β* | *t* | 95% *CI* |
| --- | --- | --- | --- | --- | --- | --- |
| Physical aggression | VVGE | 0.19 | 31.49^***^ | 0.18 | 4.21^***^ | (0.10, 0.27) |
|  | Disinhibition |  |  | 0.21 | 5.04^***^ | (0.13, 0.29) |
|  | VVGE × Disinhibition |  |  | -0.03 | -0.81 | (-0.11, 0.05) |
|  | Gender |  |  | 0.41 | 4.73^***^ | (0.24, 0.59) |

**2. The analysis of verbal aggression**

We conducted the PROCESS macro Model 4 of SPSS (Hayes, 2013) with all data standardized. In the model, VVGE was entered as the predictor, moral disengagement, anger and hostility as mediators, verbal aggression as the outcome variable, and gender as a covariate. The mediation effects of moral disengagement (0.02), anger (0.08) and hostility (0.02) significant (see Table 5, Table 6). Moral disengagement, anger and hostility account for 11.11%, 44.44%, and 11.11% of the total effect respectively. When controlling for moral disengagement, anger and hostility, the direct effect of VVGE on aggression was not significant (*β* = 0.06, *SE* = 0.04, 95% *CI* = [-0.01, 0.14]). Moral disengagement, anger and hostility wholly mediated the relationship between VVGE and aggression with 66.67% of the total effect.

**Table 5**. Testing the mediation effect of violent video games exposure on verbal aggression (standardized coefficient)

|  | Predictors | *R^2^* | *F* | *β* | *t* | 95% *CI* |
| --- | --- | --- | --- | --- | --- | --- |
| Model 1 | VVGE | 0.20 | 67.94^***^ | 0.14 | 3.46^***^ | (0.06, 0.23) |
| (Moral disengagement) | Gender |  |  | 0.74 | 8.90^***^ | (0.58, 0.91) |
| Model 2 | VVGE | 0.04 | 10.58^***^ | 0.21 | 4.55^***^ | (0.12, 0.30) |
| (Anger) | Gender |  |  | -0.10 | -1.12 | (-0.28, 0.08) |
| Model 3 | VVGE | 0.03 | 7.87^***^ | 0.14 | 3.11^**^ | (0.05, 0.23) |
| (Hostility) | Gender |  |  | 0.10 | 1.05 | (-0.08, 0.28) |
| Model 4 | VVGE | 0.35 | 57.17^***^ | 0.06 | 1.68 | (-0.01, 0.14) |
| (Verbal aggression) | Moral disengagement |  |  | 0.15 | 3.44^***^ | (0.06, 0.23) |
|  | Anger |  |  | 0.36 | 7.97^***^ | (0.27, 0.45) |
|  | Hostility |  |  | 0.17 | 3.70^**^ | (0.08, 0.26) |
|  | Gender |  |  | 0.08 | 0.98 | (-0.08, 0.24) |

**Table 6.** The direct effect and the mediation effect of moral disengagement, anger and hostility

|  | *ab* | *SE* | 95% *CI* |
| --- | --- | --- | --- |
| Mediation effect 1 (Moral disengagement) | 0.02 | 0.01 | (0.01, 0.05) |
| Mediation effect 2 (Anger) | 0.08 | 0.02 | (0.04, 0.12) |
| Mediation effect 3 (Hostility) | 0.02 | 0.01 | (0.01, 0.05) |
| Total indirect effect | 0.12 | 0.03 | (0.06, 0.18) |
| Direct effect | 0.06 | 0.04 | (-0.01, 0.14) |

We conducted the PROCESS macro Model 1 of SPSS with disinhibition as a moderator, VVGE as the predictor, verbal aggression as the outcome variable, and gender as a the covariate (Hayes, 2013). The results showed that the moderation effect of disinhibition was not significant (*β* = -0.03, *t* = -0.77, 95% *CI* = [-0.12, 0.05]), see Table 7.

**Table 7**. Testing the moderation effect of violent video games on aggression

| Outcome | Predictors | *R^2^* | *F* | *β* | *t* | 95% *CI* |
| --- | --- | --- | --- | --- | --- | --- |
| Verbal aggression | VVGE | 0.06 | 8.05^***^ | 0.19 | 4.09^***^ | (0.10, 0.28) |
|  | Disinhibition |  |  | 0.04 | 0.83 | (-0.05, 0.29) |
|  | VVGE × Disinhibition |  |  | -0.03 | -0.77 | (-0.12, 0.05) |
|  | Gender |  |  | 0.14 | 1.52 | (-0.04, 0.33) |

**3. The mediation and moderating effect of disinhibition**

To test the hypothesis that disinhibition would mediate the relationship between VVGE and total aggression, we conducted a test using the PROCESS macro Model 4 of SPSS (Hayes, 2013) with all data standardized. In the model, VVGE was entered as the predictor, disinhibition as a mediator, aggression as the outcome variable, and gender as a covariate. The relation between VVGE and disinhibition was not significant, and the mediation effect of disinhibition was also not significant (*ab*=0.01, *SE*=0.01, *95% CI* = [-0.004, 0.03]) (see Table 8).

**Table 8**. Testing the mediation effect of violent video games exposure on aggression

|  | Predictors | *R^2^* | *F* | *β* | *t* | 95% *CI* |
| --- | --- | --- | --- | --- | --- | --- |
| Model 1  (Disinhibition) | VVGE | 0.11 | 33.61^***^ | 0.07 | 1.56 | (-0.02, 0.16) |
|  | Gender |  |  | 0.59 | 6.80^***^ | (0.43, 0.77) |
| Model 2  (Aggression) | VVGE | 0.10 | 20.46^***^ | 0.21 | 4.76^***^ | (0.12, 0.30) |
|  | Disinhibition |  |  | 0.15 | 3.44^***^ | (0.06, 0.23) |
|  | Gender |  |  | 0.14 | 1.54 | (-0.04, 0.32) |

To test the hypothesis that disinhibition would mediate the relationship between VVGE and physical aggression, we conducted a test using the PROCESS macro Model 4 of SPSS (Hayes, 2013) with all data standardized. In the model, VVGE was entered as the predictor, disinhibition as a mediator, physical aggression as the outcome variable, and gender as a covariate. The relation between VVGE and disinhibition was not significant, and the mediation effect of disinhibition was also not significant (*ab*=0.01, *SE*=0.01, *95% CI* = [-0.001, 0.04]) (see Table 9).

**Table 9**. Testing the mediation effect of violent video games exposure on physical aggression

|  | Predictors | *R^2^* | *F* | *β* | *t* | 95% *CI* |
| --- | --- | --- | --- | --- | --- | --- |
| Model 1  (Disinhibition) | VVGE | 0.11 | 33.61^***^ | 0.07 | 1.56 | (-0.02, 0.16) |
|  | Gender |  |  | 0.60 | 6.80^***^ | (0.43, 0.77) |
| Model 2  (Physical aggression) | VVGE | 0.19 | 41.80^***^ | 0.17 | 4.14^***^ | (0.09, 0.26) |
|  | Disinhibition |  |  | 0.21 | 5.09^***^ | (0.13, 0.29) |
|  | Gender |  |  | 0.42 | 4.76^***^ | (0.24 0.59) |

To test the hypothesis that disinhibition would mediate the relationship between VVGE and verbal aggression, we conducted a test using the PROCESS macro Model 4 of SPSS (Hayes, 2013) with all data standardized. In the model, VVGE was entered as the predictor, disinhibition as a mediator, verbal aggression as the outcome variable, and gender as a covariate. The relation between VVGE and disinhibition , verbal aggression and disinhibition was not significant, and the mediation effect of disinhibition was also not significant (*ab*=0.002, *SE*=0.004, *95% CI* = [-0.002, 0.02]) (see Table 10).

**Table 10**. Testing the mediation effect of violent video games exposure on verbal aggression

|  | Predictors | *R^2^* | *F* | *β* | *t* | 95% *CI* |
| --- | --- | --- | --- | --- | --- | --- |
| Model 1  (Disinhibition) | VVGE | 0.11 | 33.61^***^ | 0.07 | 1.56 | (-0.02, 0.16) |
|  | Gender |  |  | 0.60 | 6.80^***^ | (0.43, 0.77) |
| Model 2  (Verbal aggression) | VVGE | 0.05 | 10.54^***^ | 0.18 | 4.03^***^ | (0.09, 0.27) |
|  | Disinhibition |  |  | 0.04 | 0.87 | (-0.05, 0.13) |
|  | Gender |  |  | 0.15 | 1.54 | (-0.04 0.33) |

To test the hypothesis that disinhibition would mediate the relationship between VVGE and aggressive behavior (PVagg), we conducted a test using the PROCESS macro Model 4 of SPSS (Hayes, 2013) with all data standardized. In the model, VVGE was entered as the predictor, disinhibition as a mediator, aggressive behavior as the outcome variable, and gender as a covariate. The relation between VVGE and disinhibition was not significant, and the mediation effect of disinhibition was also not significant (*ab*=0.01, *SE*=0.01, *95% CI* = [-0.001, 0.03]) (see Table 11).

**Table 11**. Testing the mediation effect of violent video games exposure on aggressive behavior

|  | Predictors | *R^2^* | *F* | *β* | *t* | 95% *CI* |
| --- | --- | --- | --- | --- | --- | --- |
| Model 1  (Disinhibition) | VVGE | 0.11 | 33.61^***^ | 0.07 | 1.56 | (-0.02, 0.16) |
|  | Gender |  |  | 0.60 | 6.80^***^ | (0.43, 0.77) |
| Model 2  (Aggressive behavior) | VVGE | 0.16 | 33.62^***^ | 0.20 | 4.67^***^ | (0.12, 0.29) |
|  | Disinhibition |  |  | 0.16 | 3.90^***^ | (0.08, 0.24) |
|  | Gender |  |  | 0.35 | 3.97^***^ | (0.18, 0.53) |

**4. The mediating effect of moral disengagement, anger and hostility**

We made a set of tests which included all four of the potential mediators simultaneously. We reported the analysis of PVagg in the main manuscript, and the other two are reported as above (see Table 2-Table 7) and as below (Table 12-Table 20).

**4.1 The analysis of physical aggression**

To test Hypothesis 1, we conducted the PROCESS macro Model 4 of SPSS (Hayes, 2013) with all data standardized. In the model, VVGE was entered as the predictor, moral disengagement as a mediator, physical aggression as the outcome variable, and gender as a covariate. The mediation effect of moral disengagement (0.06) was significant (see Table 11), with *SE* = 0.02, *95% CI* = [0.02, 0.11]. When controlling for moral disengagement, the direct effect of VVGE on physical aggression was significant (*β* = 0.13, *SE* = 0.04, *95% CI* = [0.05, 0.21]). Moral disengagement partially mediated the predicted effect of VVGE on physical aggression with 31.58% of the total effect..

**Table 12**. Testing the mediation effect of violent video games exposure on physical aggression

|  | Predictors | *R^2^* | *F* | *β* | *t* | 95% *CI* |
| --- | --- | --- | --- | --- | --- | --- |
| Model 1  (Moral disengagement) | VVGE | 0.20 | 67.94^***^ | 0.14 | 3.46^***^ | (0.06, 0.23) |
|  | Gender |  |  | 0.74 | 8.90^***^ | (0.58, 0.91) |
| Model 2  (Physical aggression) | VVGE | 0.29 | 77.47^***^ | 0.13 | 3.22^**^ | (0.05, 0.21) |
|  | Moral disengagement |  |  | 0.42 | 10.46^***^ | (0.34, 0.50) |
|  | Gender |  |  | 0.23 | 2.72^***^ | (0.06, 0.39) |

To test Hypothesis 2, we conducted the PROCESS macro Model 4 of SPSS (Hayes, 2013) with all data standardized. In the model, VVGE was entered as the predictor, anger as a mediator, physical aggression as the outcome variable, and gender was included as a covariate. The mediation effect of anger (0.12) was significant (see Table 13), with *SE* = 0.03, *95% CI* = [0.06, 0.18]. When controlling for anger, the direct effect of VVGE on physical aggression was significant (*β* = 0.07, *SE* = 0.04, *95% CI* = [0.0001, 0.14]). Anger partially mediated the predicted effect of VVGE on physical aggression with 63.16% of the total effect..

**Table 13**. Testing the mediation effect of violent video games exposure on physical aggression

|  | Predictors | *R^2^* | *F* | *β* | *t* | 95% *CI* |
| --- | --- | --- | --- | --- | --- | --- |
| Model 1  (Anger) | VVGE | 0.04 | 10.58^***^ | 0.21 | 4.55^***^ | (0.12, 0.30) |
|  | Gender |  |  | -0.10 | -1.12 | (-0.28, 0.08) |
| Model 2  (Physical aggression) | VVGE | 0.48 | 164.82^***^ | 0.07 | 1.97^*^ | (0.0001, 0.14) |
|  | Anger |  |  | 0.58 | 18.44^***^ | (0.52, 0.65) |
|  | Gender |  |  | 0.60 | 8.91^***^ | (0.47, 0.73) |

To test Hypothesis 2, we conducted the PROCESS macro Model 4 of SPSS (Hayes, 2013) with all data standardized. In the model, VVGE was entered as the predictor, hostility as a mediator, physical aggression as the outcome variable, and gender was included as a covariate. The mediation effect of hostility (0.07) was significant (see Table 14), with *SE* = 0.02, *95% CI* = [0.03, 0.11]. When controlling for hostility, the direct effect of VVGE on physical aggression was significant (*β* = 0.12, *SE* = 0.04, *95% CI* = [0.05, 0.19]). Hostility partially mediated the predicted effect of VVGE on physical aggression with 36.84% of the total effect.

**Table 14**. Testing the mediation effect of violent video games exposure on physical aggression

|  | Predictors | *R^2^* | *F* | *β* | *t* | 95% *CI* |
| --- | --- | --- | --- | --- | --- | --- |
| Model 1  (Hostility) | VVGE | 0.03 | 7.88^***^ | 0.14 | 3.11^**^ | (0.05, 0.23) |
|  | Gender |  |  | 0.10 | 1.05 | (-0.08, 0.28) |
| Model 2  (Physical aggression) | VVGE | 0.37 | 107.01^***^ | 0.12 | 3.23^**^ | (0.05, 0.19) |
|  | Hostility |  |  | 0.48 | 13.87^***^ | (0.41, 0.55) |
|  | Gender |  |  | 0.50 | 6.70^***^ | (0.35, 0.64) |

**4.2 The analysis of verbal aggression.**

To test Hypothesis 1, we conducted the PROCESS macro Model 4 of SPSS (Hayes, 2013) with all data standardized. In the model, VVGE was entered as the predictor, moral disengagement as a mediator, verbal aggression as the outcome variable, and gender was included as a covariate. The mediation effect of moral disengagement (0.05) was significant (see Table 15), with *SE* = 0.02, *95% CI* = [0.02, 0.09]. When controlling for moral disengagement, the direct effect of VVGE on verbal aggression was significant (*β* = 0.14, *SE* = 0.04, *95% CI* = [0.05, 0.22]). Moral disengagement partially mediated the predicted effect of VVGE on verbal aggression with 26.32% of the total effect..

**Table 15**. Testing the mediation effect of violent video games exposure on verbal aggression

|  | Predictors | *R^2^* | *F* | *β* | *t* | 95% *CI* |
| --- | --- | --- | --- | --- | --- | --- |
| Model 1  (Moral disengagement) | VVGE | 0.20 | 67.94^***^ | 0.14 | 3.46^***^ | (0.06, 0.23) |
|  | Gender |  |  | 0.74 | 8.90^***^ | (0.58, 0.91) |
| Model 2  (Verbal aggression) | VVGE | 0.15 | 31.43^***^ | 0.14 | 3.13^**^ | (0.05, 0.22) |
|  | Moral disengagement |  |  | 0.34 | 7.75^***^ | (0.26, 0.43) |
|  | Gender |  |  | -0.09 | -0.94 | (-0.27, 0.95) |

To test Hypothesis 2, we conducted the PROCESS macro Model 4 of SPSS (Hayes, 2013) with all data standardized. In the model, VVGE was entered as the predictor, anger as a mediator, verbal aggression as the outcome variable, and gender was included as a covariate. The mediation effect of anger (0.11) was significant (see Table 16), with *SE* = 0.03, *95% CI* = [0.06, 0.16]. When controlling for anger, the direct effect of VVGE on verbal aggression was significant (*β* = 0.08, *SE* = 0.04, *95% CI* = [0.002, 0.16]). Anger partially mediated the predicted effect of VVGE on verbal aggression with 57.89% of the total effect.

**Table 16**. Testing the mediation effect of violent video games exposure on verbal aggression

|  | Predictors | *R^2^* | *F* | *β* | *t* | 95% *CI* |
| --- | --- | --- | --- | --- | --- | --- |
| Model 1  (Anger) | VVGE | 0.04 | 10.58^***^ | 0.21 | 4.55^***^ | (0.12, 0.30) |
|  | Gender |  |  | -0.10 | -1.12 | (-0.28, 0.08) |
| Model 2  (Verbal aggression) | VVGE | 0.30 | 78.85^***^ | 0.08 | 2.01^*^ | (0.002, 0.16) |
|  | Anger |  |  | 0.51 | 13.95^***^ | (0.44, 0.58) |
|  | Gender |  |  | 0.22 | 2.83^**^ | (0.07, 0.37) |

To test Hypothesis 2, we conducted the PROCESS macro Model 4 of SPSS (Hayes, 2013) with all data standardized. In the model, VVGE was entered as the predictor, hostility as a mediator, verbal aggression as the outcome variable, and gender was included as a covariate. The mediation effect of hostility (0.06) was significant (see Table 17), with *SE* = 0.02, *95% CI* = [0.03, 0.11]. When controlling for hostility, the direct effect of VVGE on verbal aggression was significant (*β* = 0.12, *SE* = 0.04, *95% CI* = [0.04, 0.20]). Verbal partially mediated the predicted effect of VVGE on physical aggression with 33.33% of the total effect.

**Table 17**. Testing the mediation effect of violent video games exposure on verbal aggression

|  | Predictors | *R^2^* | *F* | *β* | *t* | 95% *CI* |
| --- | --- | --- | --- | --- | --- | --- |
| Model 1  (Hostility) | VVGE | 0.03 | 7.87^***^ | 0.14 | 3.11^**^ | (0.05, 0.23) |
|  | Gender |  |  | 0.10 | 1.05 | (-0.08, 0.28) |
| Model 2  (Verbal aggression) | VVGE | 0.25 | 59.58^***^ | 0.12 | 2.98^**^ | (0.04, 0.20) |
|  | Hostility |  |  | 0.45 | 11.83^***^ | (0.37, 0.52) |
|  | Gender |  |  | 0.13 | 1.55 | (-0.03, 0.28) |

**4.3 The analysis of aggressive behavior (the composite of physical aggression and verbal aggression)**

To test Hypothesis 1, we conducted the PROCESS macro Model 4 of SPSS (Hayes, 2013) with all data standardized. In the model, VVGE was entered as the predictor, moral disengagement as a mediator, aggressive behavior as the outcome variable, and gender was included as a covariate. The mediation effect of moral disengagement (0.06) was significant (see Table 18), with *SE* = 0.02, *95% CI* = [0.02, 0.11]. When controlling for moral disengagement, the direct effect of VVGE on aggressive behavior was significant (*β* = 0.15, *SE* = 0.04, *95% CI* = [0.07, 0.23]). Moral disengagement partially mediated the predicted effect of VVGE on aggressive behavior with 28.57% of the total effect.

**Table 18**. Testing the mediation effect of violent video games exposure on aggressive behavior

|  | Predictors | *R^2^* | *F* | *β* | *t* | 95% *CI* |
| --- | --- | --- | --- | --- | --- | --- |
| Model 1  (Moral disengagement) | VVGE | 0.20 | 67.94^***^ | 0.14 | 3.46^***^ | (0.06, 0.23) |
|  | Gender |  |  | 0.74 | 8.90^***^ | (0.58, 0.91) |
| Model 2  (Aggressive behavior) | VVGE | 0.29 | 73.89^***^ | 0.15 | 3.72^***^ | (0.07, 0.23) |
|  | Moral disengagement |  |  | 0.44 | 10.95^***^ | (0.36, 0.52) |
|  | Gender |  |  | 0.12 | 1.46 | (-0.04, 0.29) |

To test Hypothesis 2, we conducted the PROCESS macro Model 4 of SPSS (Hayes, 2013) with all data standardized. In the model, VVGE was entered as the predictor, anger as a mediator, aggressive behavior as the outcome variable, and gender was included as a covariate. The mediation effect of anger (0.13) was significant (see Table 19), with *SE* = 0.03, *95% CI* = [0.07, 0.20]. When controlling for anger, the direct effect of VVGE on aggressive behavior was significant (*β* = 0.08, *SE* = 0.03, *95% CI* = [0.02, 0.15]). Anger partially mediated the predicted effect of VVGE on aggressive behavior with 61.90% of the total effect.

**Table 19**. Testing the mediation effect of violent video games exposure on aggressive behavior

|  | Predictors | *R^2^* | *F* | *β* | *t* | 95% *CI* |
| --- | --- | --- | --- | --- | --- | --- |
| Model 1  (Anger) | VVGE | 0.04 | 10.58^***^ | 0.21 | 4.55^***^ | (0.12, 0.30) |
|  | Gender |  |  | -0.10 | -1.12 | (-0.28, 0.08) |
| Model 2  (Aggressive behavior) | VVGE | 0.51 | 189.30^***^ | 0.08 | 2.45^*^ | (0.02, 0.15) |
|  | Anger |  |  | 0.63 | 20.49^***^ | (0.57, 0.69) |
|  | Gender |  |  | 0.52 | 7.91^***^ | (0.39, 0.64) |

To test Hypothesis 2, we conducted the PROCESS macro Model 4 of SPSS (Hayes, 2013) with all data standardized. In the model, VVGE was entered as the predictor, hostility as a mediator, aggressive behavior as the outcome variable, and gender was included as a covariate. The mediation effect of hostility (0.08) was significant (see Table 20), with *SE* = 0.02, *95% CI* = [0.03, 0.12]. When controlling for hostility, the direct effect of VVGE on aggressive behavior was significant (*β* = 0.14, *SE* = 0.04, *95% CI* = [0.07, 0.21]). Hostility partially mediated the predicted effect of VVGE on aggressive behavior with 36.84% of the total effect.

**Table 20**. Testing the mediation effect of violent video games exposure on aggressive behavior

|  | Predictors | *R^2^* | *F* | *β* | *t* | 95% *CI* |
| --- | --- | --- | --- | --- | --- | --- |
| Model 1  (Hostility) | VVGE | 0.03 | 7.87^***^ | 0.14 | 3.11^**^ | (0.05, 0.23) |
|  | Gender |  |  | 0.10 | 1.05 | (-0.08, 0.28) |
| Model 2  (Aggressive behavior) | VVGE | 0.40 | 121.92^***^ | 0.14 | 3.75^**^ | (0.07, 0.21) |
|  | Hostility |  |  | 0.53 | 15.64^***^ | (0.46, 0.59) |
|  | Gender |  |  | 0.40 | 5.56^***^ | (0.26, 0.54) |
